# Strain Plethysmography Using a Hermetically Sealed MEMS Strain Sensor

**DOI:** 10.3390/bios15050325

**Published:** 2025-05-20

**Authors:** Xinyu Jiang, Brian Sang, Haoran Wen, Gregory Junek, Jin-Woo Park, Farrokh Ayazi

**Affiliations:** 1 School of Electrical and Computer Engineering, Georgia Institute of Technology, Atlanta, GA 30332, USA; xjiang362@gatech.edu (X.J.); bsang3@gatech.edu (B.S.); 2 StethX Microsystems Inc., Atlanta, GA 30308, USA; haoran@stethxmicro.com (H.W.); greg@stethxmicro.com (G.J.); jin@stethx.com (J.-W.P.)

**Keywords:** arterial pulse waveform, Blood Pressure, cardiovascular disease, MEMS, pulse transit time, strain sensors

## Abstract

We present a hermetically sealed capacitive microelectromechanical system (MEMS) strain sensor designed for arterial pulse waveform extraction using the strain plethysmography (SPG) modality. The MEMS strain sensor features a small form factor of 3.3 mm × 3.3 mm × 1 mm, leverages a nano-gap fabrication process to improve the sensitivity, and uses a differential sensing mechanism to improve the linearity and remove the common mode drift. The MEMS strain sensor is interfaced with an application-specific integrated circuit (ASIC) to form a compact strain sensing system. This system exhibits a high strain sensitivity of 316 aF/µε, a gauge factor (GF) of 35, and a strain sensing resolution of 1.26 µε, while maintaining a linear range exceeding 700 µε. SPG signals have been reliably captured at both the fingertip and wrist using the MEMS strain sensor with high signal quality, preserving various photoplethysmography (PPG) features. Experimental results demonstrate that heart rate (HR) and heart rate variability (HRV) can be estimated from the SPG signal collected at the fingertip and wrist using the sensor with an accuracy of over 99%. Pulse arrival time (PAT) and pulse transit time (PTT) have been successfully extracted using the sensor together with a MEMS seismometer, showcasing its potential for ambulatory BP monitoring (ABPM) application.

## 1. Introduction

Wearable devices capture various physiological signals generated during cardiovascular activities to extract features related to diseases. Common modalities include electrocardiography (ECG), which detects electrical activity from ion flux [1,2]; photoplethysmography (PPG), which measures blood volume changes in peripheral arteries [3]; and seismocardiography (SCG), which senses heart-induced vibrations in the chest region [4].

PPG is a widely used non-invasive modality in clinical settings and wearable devices due to its simplicity and ability to monitor continuously. It collects the arterial pulse waveform (APW) using a light-based sensor. This APW has been extensively studied, for example, for blood pressure (BP) estimation [5] and cardiac output (CO) estimation [6]. Strain plethysmography (SPG) is another modality that extracts the arterial pulse waveform and shares the same morphological features as PPG [7]. It measures the strain variation on peripheral limbs to determine changes in blood flow volume. Initially, strain plethysmography was conducted using a cumbersome strain gauge consisting of a rubber cuff with mercury filling [8]. Recent studies have developed novel strain sensors and successfully extracted the SPG signal from the fingertip using a Fiber-Bragg Grating strain sensor [9] and from the wrist using a customized strain-gauge-based strain sensor [10]. However, further investigation is needed to reduce the form factor and optimize the sensor readout schemes to enable the practical use of these sensors in ambulatory monitoring settings. Additionally, despite recent advancements in high-sensitivity stretchable strain sensors for physiological signal monitoring [11], their limited lifespan due to polymer degradation hinders their suitability for long-term wearable sensing applications.

Microelectromechanical systems (MEMS) sensors exhibit great potential in wearable sensing applications due to their small size, low weight, low power consumption, and low cost (SWaP-C). For example, MEMS accelerometers have been used as contact microphones (ACM) to sense mechano-acoustic cardiopulmonary signals [12], and MEMS gyroscopes have been used in gyrocardiography (GCG) for myocardial deformation monitoring [13]. Piezoresistive MEMS pressure sensors have been reported for pulse waveform extractions as well; however, the pulse waveform collected from the pressure sensor was distorted and caused an error in pulse wave velocity calculation [14].

Here, we introduce a wafer-level packaged (WLP) capacitive MEMS strain sensor featuring a compact form factor of 3.3 mm × 3.3 mm × 1 mm for arterial pulse waveform extractions. This sensor has a large linear range and high sensitivity, designed for physiological strain sensing, which offers a robust and energy-efficient hardware-level solution to the skin-tone artifact issue commonly encountered in PPG-based wearable sensors for arterial pulse waveform extraction. As the artery dilation generates three-dimensional strain, the out-of-plane transduction mechanism of our sensor allows it to capture the SPG signal with greater fidelity compared to previously proposed MEMS-based strain sensors [15]. The wafer-level packaging enables hermetic encapsulation of the sensor, shielding it from corrosives like sweat, hence enhancing its repeatability and lifetime.

A highly integrated strain sensing system is built for signal sampling and transmission, including the MEMS strain sensor, an application-specific integrated circuit (ASIC) sensor interface, a thin printed circuit board (PCB), and a top metal cap to protect the wire bonding. The small form of the sensing system allows it to be easily placed on various pulse sites such as the fingertip and the wrist. It is demonstrated that the SPG signals collected closely track the PPG signals and preserve various PPG features.

The potential integration of the sensor into daily objects to facilitate wearable data collection is explored, proposing a novel method for collecting pulse signals by simply holding a smartphone with a strain sensor attached to its side. This innovative approach leverages everyday interactions with mobile devices, enabling pulse monitoring without needing dedicated equipment, making it an accessible solution for continuous health tracking.

We also investigate the application and relevance of the SPG signal in cardiovascular parameter extraction and estimation. Heart rate (HR) and heart rate variability (HRV) are derived from SPG signals collected consistently from users’ fingertips and wrists. Additionally, we demonstrate the mounting of the SPG sensor on the side of a smartphone and the acquisition of HR and HRV while being held by a user. The strain sensor is also used synchronously with a MEMS seismometer [12] to form a multimodality sensing platform with a 40 µs timing resolution and a synchronous output. Using this platform, we successfully extracted pulse arrival time (PAT) and pulse transit time (PTT) to demonstrate the potential of the sensor for continuous cuffless BP monitoring [16,17].

## 2. Materials and Methods

### 2.1. Strain Sensor Design

**Figure 1:**
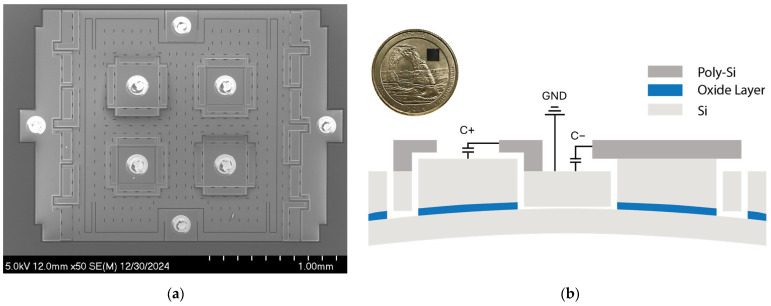
Capacitive MEMS strain sensor: (**a**) SEM image of the MEMS strain sensor. (**b**) Cross-section of the MEMS sensor showing the differential capacitive sensing pair. C+ and C− share a common terminal connected to the ground. The inset shows the wafer-level-packaged MEMS strain sensor die placed on a US quarter.

Figure 1a shows the scanning electron microscopy (SEM) image of the MEMS strain sensor built on a silicon-on-insulator (SOI) substrate [18], which consists of a suspended silicon plate loosely coupled to the Si handle layer through two folded beam springs, where the ends of the springs are anchored to the handle layer via the buried oxide layer of an SOI substrate. The sensor resolves the input strain through axial compression and vertical deflection. The design utilizes four variable capacitors for transduction with a nominal transduction gap $d_{0}$ of 200 nm. The variable capacitors are configured to form a differential sensing pair, C+ and C−. For example, in Figure 1b, when the strain is compressive, the anchors move upwards with the handle layer due to vertical deflection while the suspended plate remains flat. This causes a gap change between the poly-Si electrodes and the Si device layer. The gap change causes the capacitance output C+ to increase and C− to decrease. This differential sensing mechanism improves the linearity of the strain sensor by removing the second-order non-linear component:

(1) $$\Delta C_{out}=\frac{\epsilon_{0}A}{d_{0}-\delta d}-\frac{\epsilon_{0}A}{d_{0}+\delta d}=\frac{2\epsilon_{0}A}{d_{0}^{2}}\delta d+O\left(\delta d^{3}\right),$$

where $\epsilon_{0}$ is the electric permittivity of the air, $d_{0}$ is the nominal gap size of the variable capacitors with zero input strain, δd is the capacitor gap change due to the input strain, and A is the area of the variable capacitor. The differential sensing mechanism also removes common-mode drifts.

**Figure 2:**
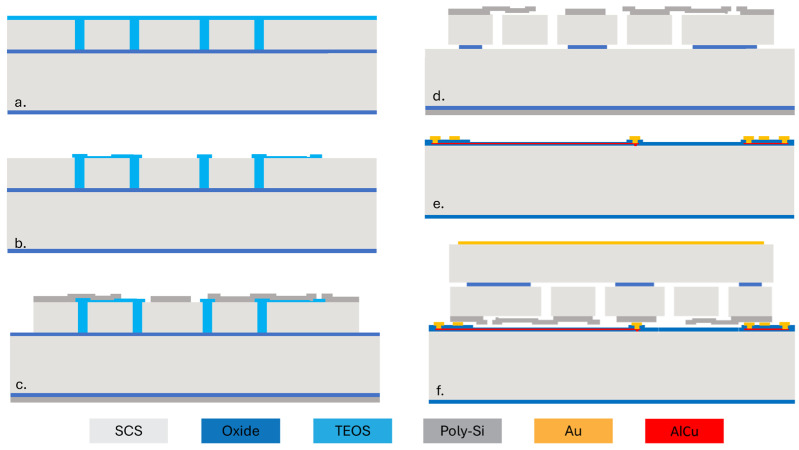
Fabrication process flow for the capacitive MEMS strain sensor. (**a**) The SOI device layer is patterned and filled with TEOS. (**b**) Pattern TEOS. (**c**) Deposit and pattern poly-Si. (**d**) Oxide etching to release the suspended plate. (**e**) Prepare the cap wafer. (**f**) Wafer bonding and stealth dicing.

Figure 2 shows the process flow of the capacitive MEMS strain sensor. First, a silicon-on-insulator (SOI) wafer is etched using deep reactive ion etching (DRIE) to create trenches defining the structure. The trenches are subsequently filled with low-pressure chemical vapor deposition (LPCVD) tetraethyl orthosilicate (TEOS), as shown in Figure 2a. The oxide on the top surface is then patterned to define a 200 nm air gap for the variable capacitor (Figure 2b). Then, a layer of LPCVD poly-Si is deposited on top of the oxide and patterned. After that, a vapor hydrogen fluoride (VHF) etching of sacrificial SiO2 is performed to release the suspended plate (Figure 2d). Figure 2e shows the cap wafer prepared through oxidation, patterning, and metal electroplating (Figure 2e). Then, the cap wafer and the device wafer are bonded together using an Au-Si eutectic bond process. The handle layer of the wafer is ground and is metalized for electric connection. Finally, stealth dicing reveals the wire bonding pad and singulates the dies (Figure 2f).

**Figure 3:**
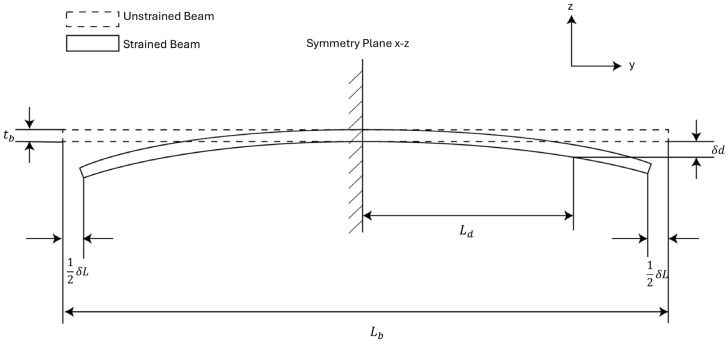
Analytical model for strain sensors. The strain sensor is treated as a prismatic beam with a central symmetric plane.

We developed a theoretical model for analyzing the strain sensitivity of the MEMS sensor based on this cross-sectional view to help the sensor design, as shown in Figure 3. In the model, the sensor is approximated as a prismatic beam for simplicity. We can view the sensor to be symmetric about the x–z plane. Under this assumption, there will be no displacement along the *z*-axis at the symmetric plane. We can impose the first boundary condition on the system by choosing the cross-section of the beam on the symmetric plane as a fixed plane.

In Figure 3, the thickness of the beam $t_{b}$ is the sum of the handle layer thickness of the sensor and the device layer thickness of the sensor. The length of the beam $L_{b}$ is the length of the MEMS die. The terminals of the variable capacitors are positioned at a distance $L_{b}$ from the symmetry plane, and the vertical displacement at the terminals is denoted as δd. The external strain is represented by prescribing a $\frac{\delta L}{2}$ displacement to the bottom edge of both sides of the beam. This gives us the second boundary condition of the system. The input strain ϵ to the beam is then represented as:

(2) $$\epsilon =\frac{\delta L}{L_{b}}.$$

### 2.2. Strain Sensing System

**Figure 4:**
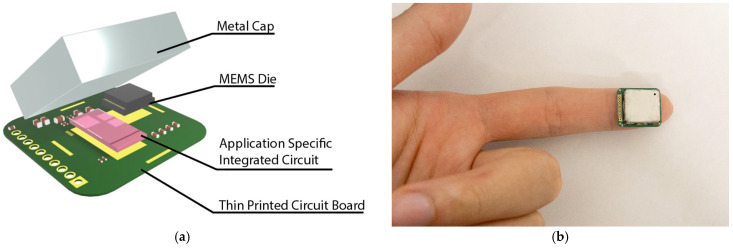
Strain sensing system: (**a**) The strain sensing system includes the MEMS die, an application-specific integrated circuit, a thin printed circuit board, and a metal cap. The complete strain sensing system features a form factor of 15 mm × 17 mm × 3 mm. (**b**) An image showing the small form sensor placed on the fingertip.

A strain sensing system is developed to read the capacitance output of the MEMS strain sensor, as shown in Figure 4a. The strain sensor is interfaced with an ASIC with a 14-bit digital output. The ASIC and the strain sensor are placed on a thin PCB and wire-bonded together. The small form of the total assembly enables it to be easily placed at the fingertips and wrist for pulse signal collection (Figure 4b). A customized cable connected to the through-hole pins is used to transmit the ASIC data to an external signal processing hub. The signal processing hub then transmits the data to a personal computer for further signal processing. The power consumption for the entire sensing system is ~200 µW.

## 3. Results and Discussion

### 3.1. Strain Sensor Finite Element Simulation

Finite element simulations (FEM) using COMSOL 6.1 are performed to evaluate the strain sensitivity of the MEMS sensor. The simulation utilized the solid mechanics physics interface coupled with the electrostatic physics interface to model the capacitance change induced by input strain. The simulation setup follows the analytical model discussed in the previous section, where strain is applied by prescribing a displacement to the bottom edges of the sensor, and the symmetry plane is treated as a fixed boundary condition, as shown in Figure 3. The capacitance change ΔC is simulated using the electrostatic physics interface embedded in COMSOL.

**Figure 5:**
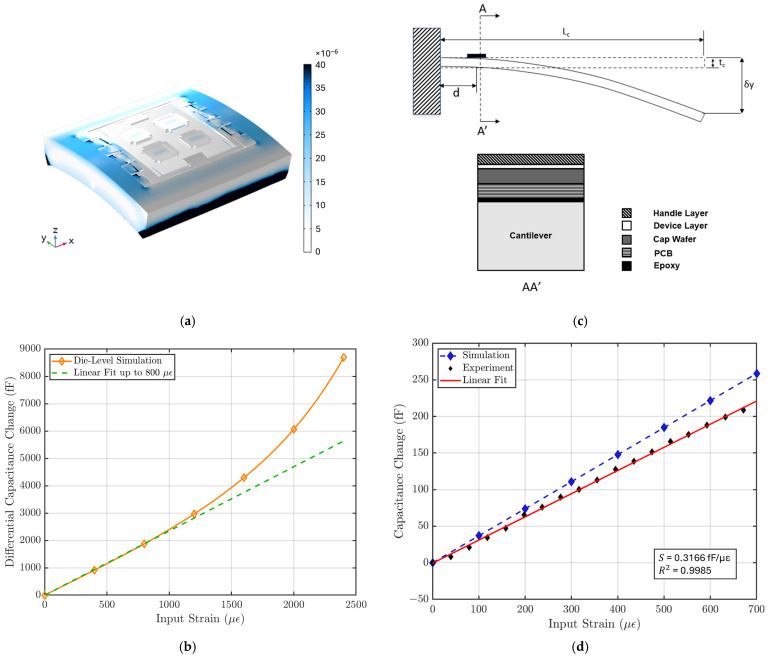
(**a**) COMSOL simulation of strain distribution in the strain sensor. (**b**) Die-level FEM simulation result shows a strain sensitivity of 2.34 fF/µε with a linear range of 800 µε. (**c**) Strain sensitivity characterization test setup. The strain sensor is placed near the fixed end of a long cantilever, and the input strain is controlled by the displacement δy at the free end of the cantilever using a micrometer. The cross-section AA’ is shown on the right [18]. (**d**) The characterization result is compared with FEM simulation result using the cantilever setup specifications. The strain sensor system shows a sensitivity of 316 aF/µε, a linear range of 700 µε, and a linearity of 99.85%.

Figure 5a illustrates that the strain is primarily concentrated in the handle layer of the sensor, while the suspended plate experiences minimal strain. Figure 5b shows the relationship between capacitance output and input strain for the MEMS sensor, with a linear fit applied to estimate the strain sensitivity within the sensor’s linear operating range. This die-level simulation shows that the MEMS strain sensor exhibits a strain sensitivity of 2.34 fF/µε and a linear range of 800 µε. The strain sensitivity S of the sensor is calculated using:

(3) $$S=\frac{\Delta C}{\epsilon }.$$

where ΔC is the capacitance change and *ε* is the input strain. The simulation also shows the sensor has a nominal capacitance $C_{0}$ of 8.8 pF. The gauge factor (GF) of the sensor is calculated to be 265 using:

(4) $$\mathrm{GF}=\frac{\Delta C}{\epsilon C_{0}}.$$

**Figure A1:**
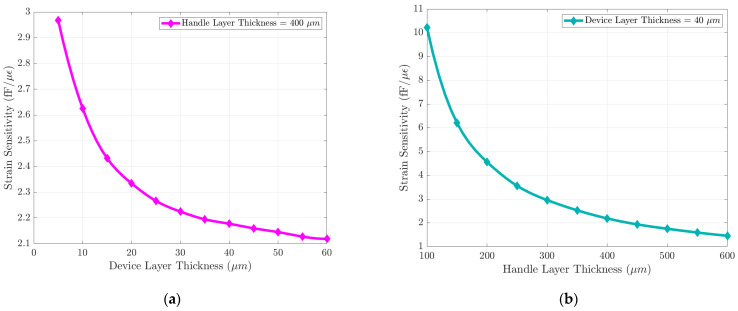
Die level FEM parametric simulation describing effects of the device layer and the handle layer thickness on strain sensor sensitivity: (**a**) The strain sensor sensitivity improves slightly with a thinner device layer (5–60 µm). (**b**) The strain sensor sensitivity can be improved substantially by reducing the thickness of the handle layer (100–600 µm).

The impact of design parameters on the strain sensitivity was also studied parametrically. The prescribed displacement at the edge of the MEMS die shown in Figure 3 is accounted for by a combination of lateral compression and bending of the sensor. For a simple beam, the lateral spring constant is proportional to the beam thickness $t_{b}$_,_ and the bending spring constant is proportional to $t_{b}^{3}\;$ [19]. The strain sensitivity can be improved substantially by reducing the handle layer thickness of the MEMS die. Figure A1 shows the FEM parametric simulation of the handle layer and the device layer thickness. In this work, the strain sensor was implemented in a SOI wafer with a 40 µm-thick device layer to be compatible with the process flow of an out-of-plane seismometer used for cardiovascular SCG monitoring as described later in this paper. A comparison between our design and other strain sensor designs is presented in Table 1.

### 3.2. Strain Sensing System Characterization

#### 3.2.1. Strain Sensitivity Characterization

The silicon MEMS die has a significantly higher Young’s modulus compared to the PCB. This causes less strain to be coupled from the test surface to the MEMS sensor and reduces the strain sensitivity of the sensing system. This requires us to conduct a strain sensitivity characterization on the complete sensing system. As shown in Figure 5c, a cantilever beam setup was used to characterize the strain sensitivity. The strain sensing system was bonded with epoxy near the fixed end of the cantilever beam. The strain input to the sensing system was controlled by the displacement at the end of the cantilever, following the relationship [15]:

(5) $$\epsilon =\frac{3t_{c}\delta y\left(L_{c}-d\right)}{2L_{c}^{3}},$$

where $L_{c}$ is the length of the cantilever, $t_{c}$ is the thickness of the cantilever, δy is the displacement at the end, which is controlled by a micrometer, and d is the distance of the sensor to the end of the cantilever beam.

**Figure A2:**
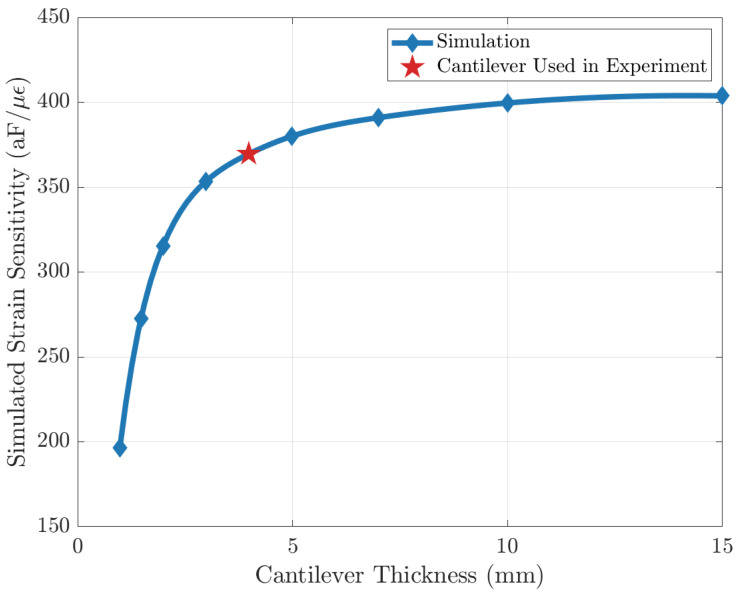
Simulation result of strain sensitivity with different cantilever thicknesses. The error is due to the loading of the strain sensor being less than 10% when the thickness of the cantilever beam is 4 mm.

Since the strain sensing system loads the cantilever beam and causes deviations from theoretical behavior in Equation (5), a FEM simulation is performed to characterize the effect of this loading. In the simulation, the entire strain-sensing system is modeled and placed at the end of a cantilever, and the strain is introduced by the displacement at the other end, as shown in Figure 5c. It is found that strain sensitivity approaches an asymptote as cantilever beam thickness increases, showing that the effect of loading becomes less significant (Figure A2). To optimize the strain input error and the loading-effect error, a cantilever beam thickness $t_{c}$ of 4 mm is used for characterization.

The characterization result is compared with FEM simulation result with the same cantilever setup. Figure 5d shows that the strain sensing system has a strain sensitivity of 316 aF/µε and a gauge factor of 35 with a linear range of 700 µε, where the nominal capacitance is measured to be ~2.2 pF for each variable capacitor using an Agilent E4980A Precision LCR Meter (Keysight, Santa Rosa, CA, USA). The non-linear behavior during the characterization is not limited by the MEMS sensor but by the ASIC.

#### 3.2.2. Response to Dynamic Loads

**Figure 6:**
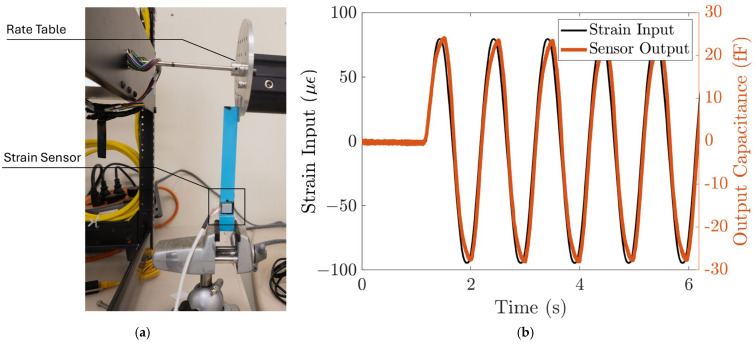
The strain sensing system responds to dynamic input strain. (**a**) Test setup. The strain input to the strain sensor is controlled using a rate table. (**b**) The sensor output closely follows the dynamic input strain with ~300 aF/µε sensitivity and high repeatability.

The response of the strain sensing system to dynamic loads is characterized using the strain induced by a rate table. The strain sensing system is first bonded to the cantilever beam, similar to the static characterization setup. Then, the end of the cantilever beam is attached to the edge of the rate table, clamped by two metal plates, as shown in Figure 6a. A sinusoidal function is commanded to the rate table, with an amplitude of 0.36 degrees and a frequency of 1 Hz. The strain input to the sensing system can be calculated from the displacement using (5), and the displacement at the end of the cantilever, δy, is the radius of the rate table multiplied by the angular position of the rate table. The angular position of the rate table is read by the rate table’s embedded sensor. This corresponds to a sinusoid strain input with an amplitude of 78 µε. We can then plot the input strain as a function of time. Figure 6b shows that the strain sensor output closely follows the strain input with high repeatability. The strain sensing system’s sensitivity to the dynamic input strain is ~300 aF/µε, which agrees with the static characterization result.

**Figure 7:**
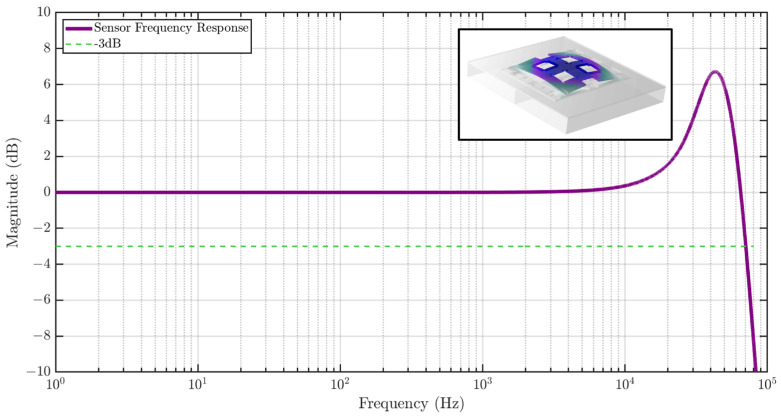
Frequency response of the MEMS strain sensor to strain input simulated using FEM analysis. The sensor exhibits a high bandwidth of 65 kHz. The inset shows the first eigenmode of the sensor at 46.3 kHz.

The sensor frequency response is also studied using COMSOL as shown in Figure 7, showing a flat gain bandwidth of greater than 10 kHz. A Q of 10 was assumed in the simulation, which causes peaking in the frequency response. The inset shows the first eigenmode of the strain sensor at 46.3 kHz.

#### 3.2.3. Resolution

**Figure 8:**
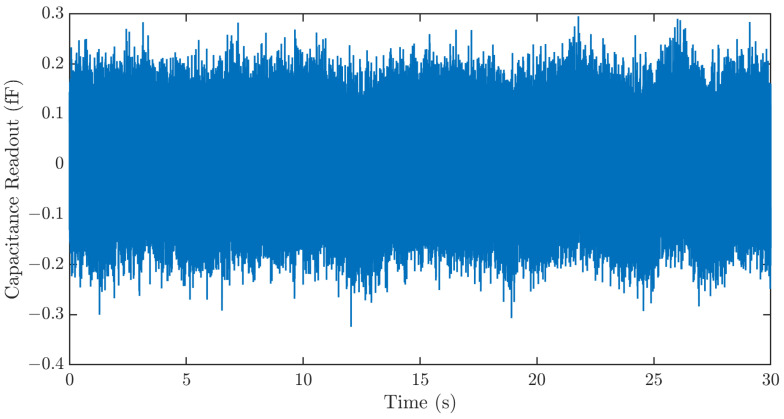
Time series noise output of the strain sensing system. The sensing system exhibits a noise of ~400 aF. This corresponds to a strain sensing resolution of 1.26 µε.

Three major noise sources, the Brownian noise of the MEMS sensor, the electronic noise of the ASIC, and quantization noise, limit the resolution of the strain sensing system. To characterize the resolution of the strain sensing system, the sensing system output is recorded for 30 s using the data processing hub without signal input. The result in Figure 8 shows a sensor noise of ~400 aF. This corresponds to a strain sensing resolution of 1.26 µε.

### 3.3. Cardiovascular Parameter Extraction

#### 3.3.1. Experimental Setup

**Figure 9:**
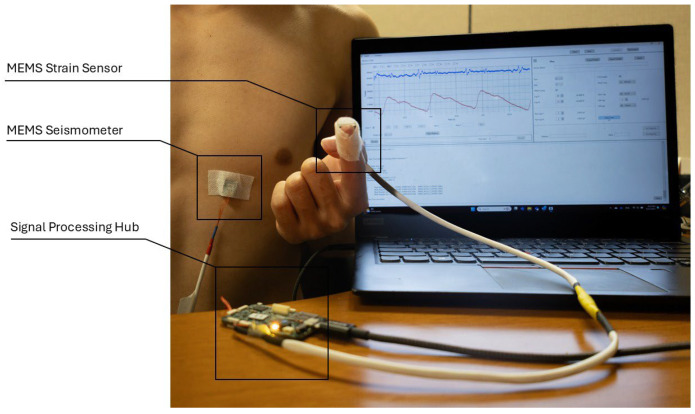
Experiment setup for cardiovascular parameter estimation. The SCG signal (blue) and SPG signal (red) collected from the sensors are sampled by the signal processing hub and displayed on the computer monitor.

We conducted four experiments to verify the MEMS strain sensor’s reliability in collecting the SPG signals. Figure 9 depicts the general setup for all four experiments, where a MEMS seismometer is used together with the strain sensor. The MEMS seismometer was placed in the tricuspid region to collect the SCG signal for all four experiments, while the strain sensor placement varied across experiments for SPG signal collection.

**Figure 10:**
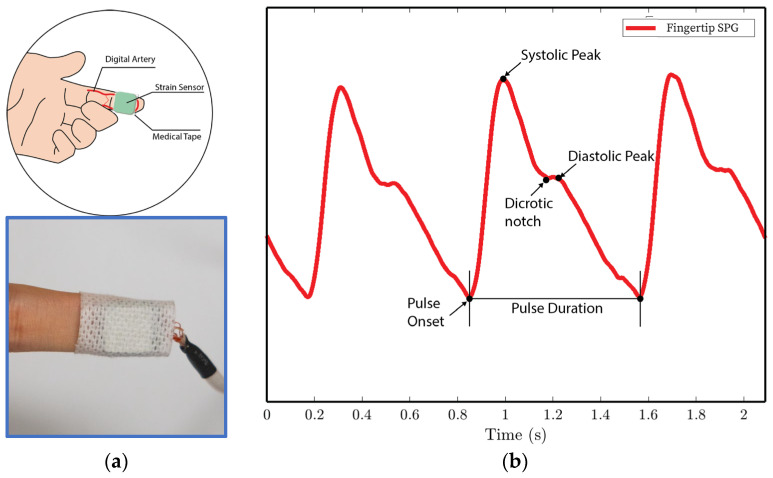
SPG signals are collected from the fingertip pulse location for experiments 1 and 4. (**a**) Illustration of sensor placement at the digital artery of the fingertip. Medical tape is used to attach the sensor to the fingertip. (**b**) SPG signal collected from the fingertip possesses the key features of the PPG signal.

**Figure 11:**
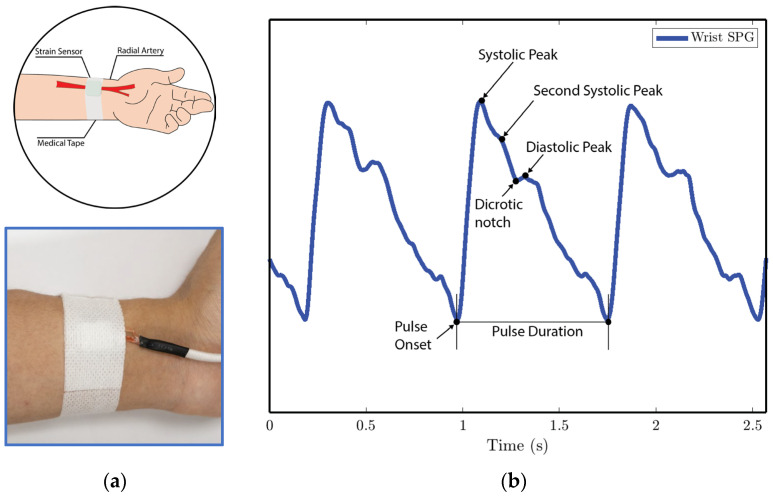
SPG signals are collected from the wrist pulse location for experiment 2. (**a**) Illustration of sensor placement at the radial artery of the wrist. Medical tape is used to attach the sensor to the wrist. (**b**) SPG signal collected from the wrist possesses key features of the PPG signal.

**Figure 12:**
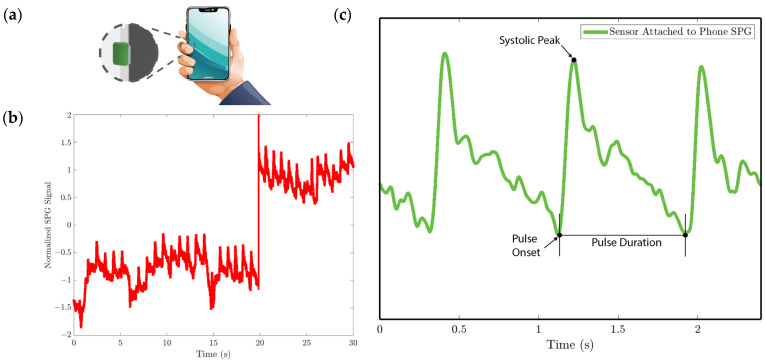
SPG signal is collected using the strain sensor attached to the cell phone for experiment 3. (**a**) Illustration of collecting the pulse signal from the digital artery at the fingertip by holding the cell phone with the strain sensor attached to the side. (**b**) Baseline drift due to the gripping force variation. (**c**) SPG signal collected preserves the shape and some features of the pulse waveform.

- In experiments 1 and 4, the MEMS strain sensor is wrapped around the fingertip using medical tape as shown in Figure 10a.
- In experiment 2, the MEMS strain sensor is placed on the wrist and fixed with medical tape, as shown in Figure 11a.
- In experiment 3, the MEMS strain sensor is first attached to the side of a cell phone using double-sided tape. The subject then holds the phone to collect the SPG signal, as shown in Figure 12a.

Throughout all experiments, both the SCG and SPG signals were recorded synchronously. The SCG signal and the SPG signal were displayed on a laptop screen during the tests, represented by the blue line and the red line in the screen monitor in Figure 9. The seismometer and the strain sensor are both sampled by the data processing hub with a sampling rate of 25 kHz.

Experiments 1, 2, and 3 were designed to evaluate the sensor’s capability to measure the SPG signal at different pulse locations with high fidelity and accuracy. The fidelity of the SPG signal was assessed by examining the preservation of the morphological features of the arterial pulse waveform. The collected signals are then processed and used to extract heart rate and heart rate variability. The temporal accuracy of the SPG signal was determined by comparing the HR and HRV extracted from the SPG modality with those derived from the SCG modality.

Experiment 4 aims to test the sensor’s capability of measuring pulse arrival time and pulse transit time. This experiment involved comparing the PAT/PTT under normal and elevated blood pressure conditions. The subject first rested for 3 min to collect signals under the normal BP condition. Then, after performing a 3 min stair-climbing exercise to elevate blood pressure, a second set of signals was collected to represent the signals under elevated BP conditions.

#### 3.3.2. Signal Processing

The collected SPG signals are first filtered using a second-order Butterworth filter with a pass band of 0.5–25 Hz. To remove the baseline drift, the first peak detection is performed to detect the pulse onset points of the SPG signal. A piecewise linear function is constructed by connecting the pulse onset points, which represent the sensor’s baseline drift. This piecewise linear baseline is then subtracted from the filtered SPG signal to remove the baseline drift.

The SPG signal is then normalized, and a second peak detection is performed to identify the systolic peak of the SPG signal, which will be used for cardiovascular parameter estimation. Another modality, acceleration plethysmography (APG), is also generated from the normalized SPG signal by taking the first time derivative of the SPG signal. The APG signal is an indicator of vascular aging [9]. It is also useful for HR and HRV estimation when the SPG signal is corrupted with pressure artifacts in experiment 3, and the systolic peaks could not be accurately identified using a peak detection algorithm.

**Figure 13:**
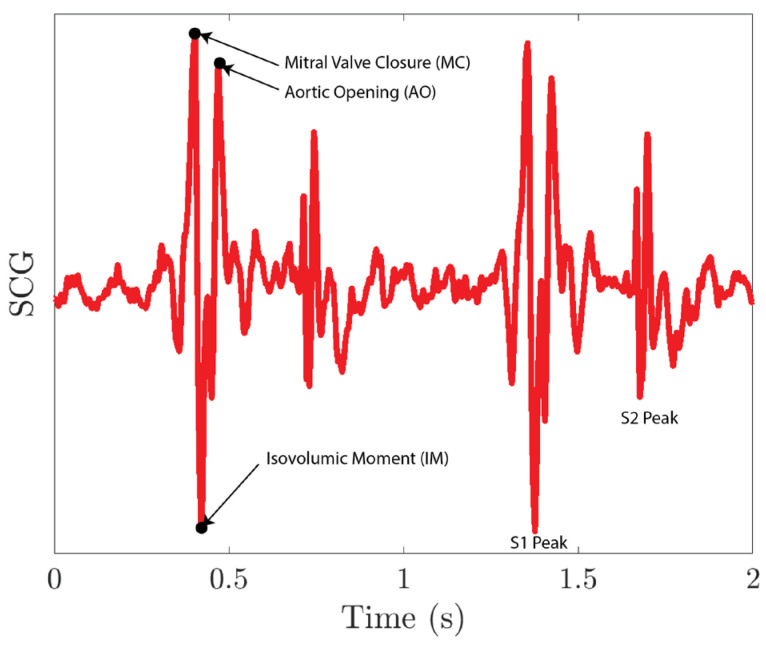
SCG signal collected using the MEMS seismometer.

The collected SCG signals are first filtered using a second-order Butterworth filter with a pass band of 0.5–80 Hz and then normalized. As shown in Figure 13, the S1 peak of the SCG signal includes a mitral valve closure peak (MC), an aortic opening peak (AO), and an isovolumic moment point (IM). As MC and AO have similar amplitudes, it is difficult for the peak detection algorithm to distinguish between these two peaks. To resolve the issue, we first run a peak detection on IM peaks. The IM peaks sit between the MC peak and the AO peak for each S1 peak. We then segment the SCG signals into single heartbeat segments using IM peaks. For each segment, we lower the threshold of the peak detection so that it captures both MC and AO peaks. The first peak location for each segment is the AO peak of the previous heartbeat, and the last peak location for each segment is the MC peak for the current heartbeat.

Heart rates are extracted from the peak-to-peak interval (PPI) of the systolic peak for the SPG signal, the peak of the APG signal, and the AO peaks of the SCG signal. The performance of the HR estimation using the SPG signal and the APG signal is evaluated by using the SCG signal as the reference signal. We also use the APG signal as a reference to the SPG signal to assess the accuracy of the SPG signal, which provides insight into how accurately the SPG signal represents the true pulse waveform, as the max slope point of the SPG signal is the peak of the APG signal. Average HR (AVG), mean absolute error (MAE), mean error (ME), standard deviation of the error (SD), and root mean square error (RMSE) are calculated to evaluate the performance of the HR estimation. The accuracy (ACC) is calculated using normalized RMSE using:

(6) $$\mathrm{ACC}=100\frac{\mathrm{AVG}-\mathrm{RMSE}}{\mathrm{AVG}}$$

Three different HRVs are then calculated using the time domain root mean square of successive differences (RMSSD) method from the estimated HR. The accuracy of HRV estimation is calculated using:

(7) $$\mathrm{ACC}=100(1-2\left|\frac{{\mathrm{HRV}}_{1}-{\mathrm{HRV}}_{2}}{{\mathrm{HRV}}_{1}+{\mathrm{HRV}}_{2}}\right|)$$

where HRV_1_ is calculated from the test modality, and HRV_2_ is calculated from the reference modality. PAT is extracted between the systolic peak of the SPG signal and the MC peak of the S1 peak of the SCG signal, and PTT is extracted between the systolic peak of the SPG signal and the AO peak of the S1 peak of the SCG signal [17]. The mean and standard deviation of the PAT and PTT are also reported.

#### 3.3.3. Cardiovascular Parameter Extraction Results

We analyze the morphological features of the collected SPG signal compared to PPG signals. Key features of PPG signals include the pulse onset point, the systolic peak, the diastolic peak, the max slope point, and the dicrotic notch [24]. Due to wave reflection, PPG signals also possess features such as the second systolic peak [25]. Preserving these features with high fidelity plays a crucial role in machine learning (ML) based cardiovascular monitoring applications [26]. Figure 10b shows the SPG signal collected from the fingertip, and Figure 11b shows the SPG signal collected from the wrist. Both pulse waveforms preserve morphological features, demonstrating that the SPG signal can be collected using the MEMS strain sensor with high fidelity. Figure 12b shows the raw signal collected from experiment 3. The sensor’s output is susceptible to pressure artifacts due to the gripping force variance. However, as shown in Figure 12c, the SPG signal collected from the sensor attached to a cell phone still preserves the distinct shape of the arterial pulse waveform.

**Figure 14:**
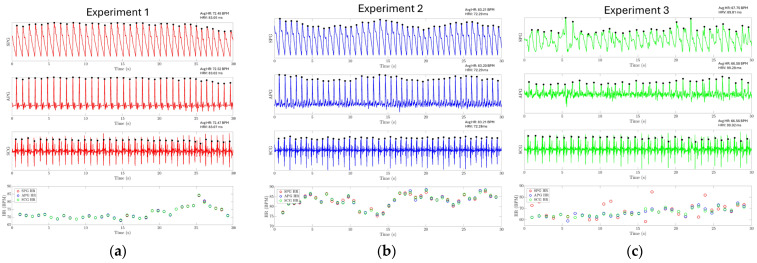
Experiment results for HR and HRV estimation. The HR and HRV for each experiment are reported on the top right of each subplot for each modality. (**a**) HR estimation using the SPG signal collected from the fingertip for experiment one. (**b**) HR estimation using the SPG signal collected from the wrist for experiment 2. (**c**) HR estimation using the SPG signal collected from the strain sensor attached to the cell phone.

The results of experiments 1, 2, and 3 are shown in Figure 14a–c, respectively. The SPG and SCG signals are plotted continuously for 30 s. For experiments 1 and 2, it is found that the HR extracted from three different modalities closely follow each other, where the mean absolute error (MAE) between SPG-APG, SPG-SCG, and APG-SCG is 0.117, 0.145, and 0.234 beats per minute (BPM) for experiment 1. The MAE between SPG-APG, SPG-SCG, and APG-SCG is 0.52, 0.653, and 0.31 BPM for experiment 2. In experiment 3, the SPG signal is distorted due to pressure artifacts. This hinders the identification of the systolic peaks from the SPG signal and reduces the accuracy of estimating HR directly from the SPG signal, with an MAE of 4.32 BPM between the SPG and SCG. However, the APG modality still has a high accuracy with an MAE of 1.35. This shows that while the systolic peaks are distorted, the SPG signal still reliably captures the maximum slope point of the arterial pulse waveform. The evaluation metrics for HR are reported in Table 2. HRV has also been estimated using the SPG with high accuracy, as reported in Table 3. These results demonstrate that SPG signals can be reliably collected from the fingertip (Experiment 1), the wrist (Experiment 2) using the MEMS strain sensor, and by holding a cell phone with the sensor attached (Experiment 3). The collected signals exhibit exceptional quality, high temporal accuracy, and precise cardiovascular information.

**Figure 15:**
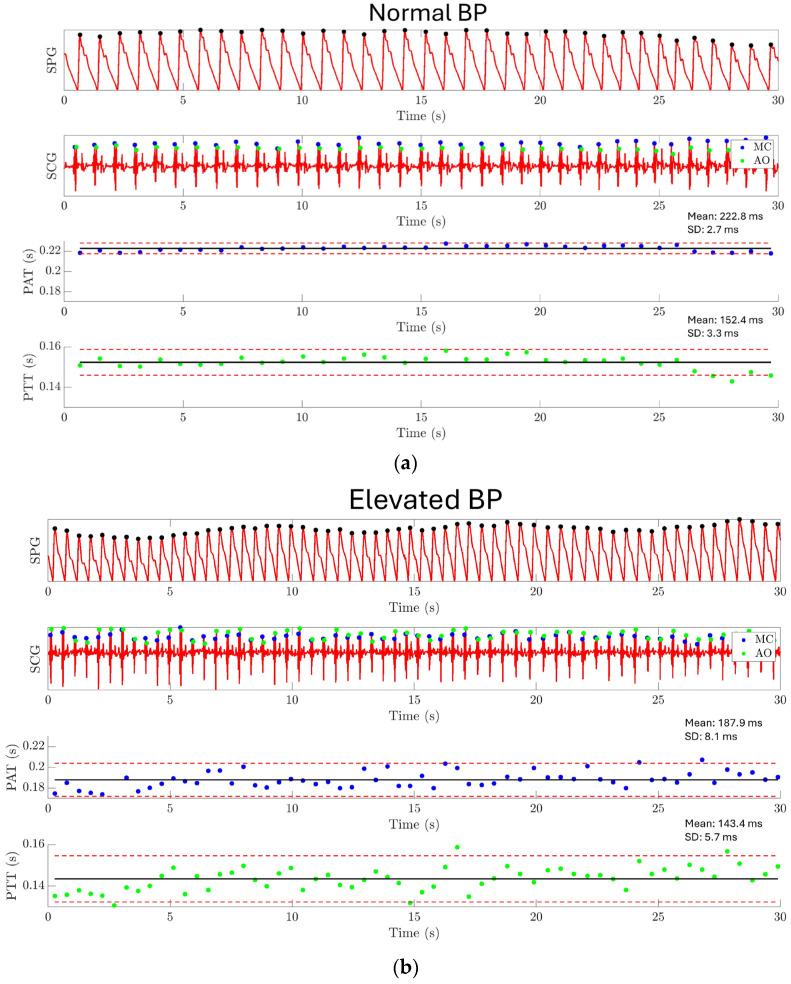
Experiment 4: Pulse arrival time and pulse transit time estimation results. (**a**) PAT and PTT are extracted under the normal BP condition. PAT has a mean value of 222.8 ms with a standard deviation of 2.7 ms, and PTT has a mean value of 152.4 ms with a standard deviation of 3.3 ms. (**b**) PAT and PTT are extracted under the elevated BP condition. PAT has a mean value of 187.9 ms with a standard deviation of 8.1 ms, and PTT has a mean value of 143.4 ms with a standard deviation of 5.7 ms. The result shows a significantly shorter PAT and PTT for the elevated BP condition compared to the normal BP condition.

The result of experiment 4 is shown in Figure 15. For normal BP conditions, PAT has a mean value of 222.8 ms with a standard deviation of 2.7 ms, and PPT has a mean value of 152.4 ms with a standard deviation of 3.3 ms, as shown in Figure 15a. Under elevated BP conditions, PAT has a mean value of 187.9 ms with a standard deviation of 8.1 ms, and PTT has a mean value of 143.4 ms with a standard deviation of 5.7 ms, as shown in Figure 15b. A significant difference has been found between the PAT/PTT values under the normal BP condition and the elevated BP condition. This result demonstrates the capability of using the MEMS strain sensor together with a MEMS seismometer for monitoring the PAT and PTT.

## 4. Conclusions

We introduced a capacitive hermetically sealed MEMS strain sensor with high sensitivity, wide linear range, and high durability for continuous monitoring of cardiovascular parameters using the SPG modality. The arterial pulse waveform has been reliably extracted from both the fingertip and wrist using the SPG, demonstrating strong agreement with the signal extracted using PPG. Heart rate and heart rate variability are also accurately estimated from the arterial pulse waveform. This further proves the high temporal resolution and high fidelity of the SPG signal collected using the strain sensor.

The strain sensor is also used in conjunction with a MEMS seismometer to form a multimodality sensing platform that collects the SPG and the SCG signal synchronously. PAT and PTT are successfully extracted using this sensing platform. The synchronous acquisition of SPG and SCG signals enhances its capability for multimodal physiological monitoring. This could play a crucial role in cardiovascular signal acquisition under a wearable setup, supporting ambulatory BP monitoring and other continuous monitoring tasks, thereby aiding in the management of chronic conditions and cardiovascular diseases.

For future studies, we are exploring a new flexible PCB design that integrates a Bluetooth module, removing the need for a separate data processing hub. This eliminates cables and enables more seamless wearable data collection.

## Figures and Tables

**Table 1:** Comparison between this work and reported strain sensors.

	Mechanism	Material	Linear Range	Scale Factor	Gauge Factor
This work	Capacitive	Si	800 µε	2.34 fF/µε	293
[15]	Capacitive	Si	1000 µε	265 aF/µε	602
[20]	LC Resonant	Si	1000 µε	34 kHz/με	430
[21]	Piezoresistive	SU-8-Graphene	50,000 µε	NA	19.1
[22]	Piezoresistive	Silver NW on PDMS	20,000 µε	NA	289
[23]	Piezoresistive	MoS_2_	7000 µε	NA	102

**Table 2:** Performance Evaluation of Heart Rate Estimation.

	SPG-APG				SPG-SCG				APG-SCG			
	MAE	ME ± SD	RMSE	ACC%	MAE	ME ± SD	RMSE	ACC%	MAE	ME ± SD	RMSE	ACC%
Fingertip	0.117	−0.03 ± 0.14	0.145	99.80	0.194	0.016 ± 0.24	0.234	99.68	0.225	−0.047 ± 0.28	0.281	99.61
Wrist	0.520	−0.01 ± 0.66	0.653	99.22	0.533	−0.012 ± 0.63	0.626	99.25	0.31	0.004 ± 0.40	0.394	99.52
Phone	4.32	1.17 ± 6.41	6.4176	90.36	4.28	1.18 ± 6.37	6.38	90.41	0.99	−0.019 ± 1.38	1.35	97.96

The performance of HR is evaluated using a second modality as the ground truth, for example, SPG-SCG uses SCG as a reference to assess the accuracy of HR estimated by SPG. SPG: Strain Plethysmography APG: Accelerated Plethysmography SCG: Seismocardiography. Unit: BPM.

**Table 3:** Accuracy of Heart Rate Variability Estimation.

	SPG-APG	SPG-SCG	APG-SCG
Fingertip	99.95	99.98	99.93
Wrist	99.99	99.99	99.99
Phone	99.21	99.28	99.92

This table reports the accuracy of HRV estimation calculated using (7).

## Data Availability

The data and code are available on https://github.com/xjiang362/Strain-Sensor-Data (accessed on 1 August 2024).
